# *FlbZIP12* gene enhances drought tolerance via modulating flavonoid biosynthesis in *Fagopyrum leptopodum*

**DOI:** 10.3389/fpls.2023.1279468

**Published:** 2023-10-11

**Authors:** Anhu Wang, Yu Liu, Qiujie Li, Xiaoyi Li, Xinrong Zhang, Jiao Kong, Zhibing Liu, Yi Yang, Jianmei Wang

**Affiliations:** ^1^ Panxi Crops Research and Utilization Key Laboratory of Sichuan Province, Xichang University, Xichang, China; ^2^ Key Laboratory of Bio-resource and Ecoenvironment of Ministry of Education, College of Life Sciences, Sichuan University, Chengdu, China

**Keywords:** *Fagopyrum leptopodum*, drought stress, flavonoids, ABA signaling, bZIP transcription factor

## Abstract

Karst lands provide a poor substrate to support plant growth, as they are low in nutrients and water content. Common buckwheat (*Fagopyrum esculentum*) is becoming a popular crop for its gluten-free grains and their high levels of phenolic compounds, but buckwheat yields are affected by high water requirements during grain filling. Here, we describe a wild population of drought-tolerant *Fagopyrum leptopodum* and its potential for enhancing drought tolerance in cultivated buckwheat. We determined that the expression of a gene encoding a Basic leucine zipper (bZIP) transcription factor, *FlbZIP12*, from *F. leptopodum* is induced by abiotic stresses, including treatment with the phytohormone abscisic acid, salt, and polyethylene glycol. In addition, we show that overexpressing *FlbZIP12* in Tartary buckwheat (*Fagopyrum tataricum*) root hairs promoted drought tolerance by increasing the activities of the enzymes superoxide dismutase and catalase, decreasing malondialdehyde content, and upregulating the expression of stress-related genes. Notably, *FlbZIP12* overexpression induced the expression of key genes involved in flavonoid biosynthesis. We also determined that FlbZIP12 interacts with protein kinases from the FlSnRK2 family *in vitro* and *in vivo*. Taken together, our results provide a theoretical basis for improving drought tolerance in buckwheat via modulating the expression of *FlbZIP12* and flavonoid contents.

## 1 Introduction

The *Fagopyrum* genus, belonging to the Polygonaceae family, is an annual or perennial, herbaceous or semi-shrub dicotyledonous plant (Zhang et al., 2017; Fan et al., 2021). Common buckwheat (*F. esculentum*) is widely cultivated in Asia, Europe, and the Americas, whereas Tartary buckwheat (*F. tataricum*) and tall buckwheat (*F. cymosum*, also known as *F. dibotrys*) are mainly cultivated in China (Li et al., 2022). In recent years, buckwheat has become a popular crop for its gluten-free grains and their high levels of phenolic compounds, thus making it suitable for people suffering from celiac disease and other gluten sensitivities (Siracusa et al., 2017).

Compared to other crops such as maize (*Zea mays*), rice (*Oryza sativa*), and wheat (*Triticum aestivum*), buckwheat is not widely cultivated (Aubert et al., 2020), and has low production (1700–2500 kg hm^−2^) (Kreft et al., 2020). The grain yields achieved by current buckwheat varieties thus cannot meet the increasing market demand. Buckwheat is widely planted in mountainous areas (Park et al., 2011), including in southwestern China with less precipitation than in other parts of the country. The high water requirement during seed maturation of buckwheat poses a severe threat to yield.

Plants have evolved sophisticated strategies to cope with drought stress. Indeed, accumulation of the phytohormone abscisic acid (ABA) triggers multiple physiological responses through ABA signaling (Soma et al., 2023), such as inhibiting seed germination and promoting stomatal closure to decrease transpiration. In addition, plants produce a variety of pigments, such as flavonoids, carotenoids, and anthocyanins (Liu et al., 2023; Mishra, 2023). These strategies are not exclusive and multiple strategies can be employed simultaneously to enhance drought tolerance.

The core components of the ABA signaling pathway comprise the ABA receptors PYRABACTIN RESISTANCE1/PYR1-LIKE/REGULATORY COMPONENTS OF ABA RECEPTOR (PYR/PYL/RCAR), type 2C protein phosphatases (PP2Cs), and sucrose non-fermenting1 (SNF1)-related protein kinase2 (SnRK2) (Ma et al., 2009; Park et al., 2009). SnRK2s phosphorylate downstream targets that include ion channels (SLOW ANION CHANNEL-ASSOCIATED1 [SLAC1] and POTASSIUM CHANNEL IN ARABIDOPSIS THALIANA1 [KAT1]) and an NADPH oxidase (RESPIRATORY BURST OXIDASE HOMOLOG F [RBOHF]), thereby regulating stomatal movement (Joshi-Saha et al., 2011). Several basic leucine zipper (bZIP) transcription factors, for example ABA-INSENSITIVE5 (ABI5), are phosphorylated by SnRK2s and then activate the expression of drought-related genes (Fujii et al., 2007). In rice, OsbZIP46 is a positive regulator of ABA signaling and overexpressing *OsbZIP46* enhances drought tolerance (Tang et al., 2012; Chang et al., 2017). The Arabidopsis bZIP-type ABF1-4 and IDD14 transcription factor form the complex to promote their transcriptional activities, and then regulate drought-stress responses (Liu et al., 2022). The ABA signaling pathway is well known in Arabidopsis (*Arabidopsis thaliana*), rice, and maize, but is rather unclear in the *Fagopyrum* genus.

Different from the cultivated buckwheat species, *F. leptopodum* is mainly distributed in rocks and in dry, hot valley areas, indicating that it may be highly drought tolerant and able to withstand stressful growth conditions (Fan et al., 2021; Li et al., 2022). Currently, only the sequence of the chloroplast genome is reported for this species (Fan et al., 2021). An investigation of the mechanisms by which *F. leptopodum* can grow in such inhospitable conditions would pave the way for the rapid identification of useful genes with direct application to crop breeding.

In order to improve the drought tolerance of cultivated buckwheat species, we investigated the wild buckwheat population. We found that the poor habitat of a wild population of *F. leptopodum* and discovered that the population is decreasing in southwestern China. Through molecular and physiological analyses, we show that the transcription factor FlbZIP12 interacts with the kinases FlSnRK2.2 and FlSnRK2.6 to confer drought tolerance in *F. leptopodum.* These findings not only elucidate the mechanism of drought tolerance in *F. leptopodum*, but provide a good start to pay attention to this species.

## 2 Materials and methods

### 2.1 Plant material and morphological analysis

The habitat of *F. leptopodum* was investigated in southwest China. Mature seeds were collected in the wild. Mature dry seeds from five populations were then sown in a greenhouse to perform morphological analysis, including inflorescence, flowers, and pollens.

### 2.2 Plant materials and treatment conditions

Seeds from Muli in **Table 1** were sown in growth chambers to analyze phenotype and perform physiological analysis. Pollen analysis was performed by scanning electron microscopy as described previously (Zhang et al., 2007). Plants in soil cultures (vermiculite: nutrient soil = 3:1) were grown in growth chambers. *F. leptopodum* seedlings were grown on Murashige and Skoog (MS) medium in a growth chamber at 25°C, under a 16-h light/8-h dark photoperiod, with 60% relative humidity, and 250 μmol photons m^−2^ s^−1^ light intensity. Ten-day-old seedlings were transferred to the MS supplemented with or without 25% (w/v) polyethylene glycol 6000 (PEG6000), 200 mM NaCl, or 50 μM ABA for 0.5, 1, 2, 3, 6, 12 or 24 h.

**Table 1:** *F. leptopodum* in this study.

Localization	Longitude	Latitude	Altitude (m)
Wenchuanli, Sichuan	103^°^34′0777″	31°30′2127″	1575.3
Libo, Sichuan	103^°^35′2255″	28^°^14′5536″	888.9
Muli, Sichuan	100^°^56′0239″	28^°^06′4367″	2503.3
Muli, Sichuan	101^°^13′2680″	27^°^46′595″	2462.5
Panzhihua, Sichuan	101^°^42′5481″	26^°^16′5844″	1592.1

### 2.3 Bioinformatics analysis

We used MEGA11 for the multiple sequence analysis and alignment as well as the phylogenetic tree analysis (Tamura et al., 2021). The Neighbor-Joining (NJ) method (1000 times) was used to construct the phylogenetic tree as previously described (Ma et al., 2019).

### 2.4 RNA extraction and RT-qPCR

Total RNA was extracted from the seedlings using an RNAout Kit (TIANGEN, China). Reverse transcription was performed with 1 µg of total RNA using Hifair^®^ II 1st Strand cDNA Synthesis SuperMix (Yeasen, Shanghai, China). QPCR was performed on a Bio-Rad real-time instrument (CFX96) with the following cycling conditions: 96°C for 2 min, 35 cycles of 96°C for 30 s and 55°C for 30 s. QPCR was performed using Hieff qPCR SYBR Green Master Mix (Yeasen, Shanghai, China). At least three repeated were performed. The relative expression levels were calculated by comparing the target genes’ cycle thresholds (CTs) with the reference gene *FlH3* (the paralog of *FtH3*, histone H3). Data quantifcation was carried out with the CFX Manager software using the 2^-ΔΔCt^ method (Darwish et al., 2022). The primers used for RT-qPCR are listed in **Table S1**.

### 2.5 Subcellular localization of FlbZIP12

The full-length coding sequence of *FlbZIP12* (1329bp) was amplified from buckwheat seedlings using primeSTAR (TaKaRa) with the following cycling conditions: 30 cycles of 98°C for 10 s, 55°C for 5 s and 72°C for 10 s. The PCR product was ligated into the pCAMBIA2300 vector to obtain pCAMBIA2300-*FlbZIP12*-GFP. The plasmids pCAMBIA2300-*FlbZIP12*-GFP and pCAMBIA2300 (negative control) were transformed into Agrobacterium (*Agrobacterium tumefaciens*) strain GV3101. Then, overnight cultures from each Agrobacterium colony were harvested and resuspended in infiltration buffer containing 0.1 mM acetosyringone, 2 mM Na_3_PO_4_, and 10 mM MES-KOH pH 5.6. The cultures were individually infiltrated into the leaves of 4-week-old *Nicotiana benthamiana* plants. At 36 h following infiltration, fluorescence signals were observed using a confocal laser scanning microscope (Leica DM4 B).

### 2.6 Induction and transformation of Tartary buckwheat hairy root cultures

Induction and transformation of *F. leptopodum* hairy roots were carried out as described previously (Yao et al., 2020). The plasmid pCAMBIA2300-*FlbZIP12*-GFP was transformed into Agrobacterium strain ACC10060, with pCAMBIA2300-GFP used as the control. Briefly, seeds were sown on half-strength solid MS medium and allowed to germinate and grow for 12 days. A 0.8–1 cm section of the hypocotyl was excised and placed on solid MS medium for 24 h. Then, the explants were soaked in an Agrobacterium culture harboring the respective plasmid for 15 min and then placed on MS medium containing 100 μM acetosyringone for 3 days in the dark. Subsequently, the explants were removed from the dark conditions and placed on hairy root induction medium containing 500 mg/L cephalosporins and 50 mg/L kanamycin for 7 days. After the hairy roots had formed, the explants were transferred to fresh medium (containing 50 mg/L kanamycin) for subculture. The hairy root cultures were incubated in the dark at 25°C on a shaker at 80 rpm.

### 2.7 Physiological measurements

Superoxide dismutase (SOD) activity, Catalase (CAT), and malondialdehyde (MDA) content measurements of buckwheat after PEG6000 were performed according to the instructions of the reagent kit (Nanjing Jiancheng Bioengineering Institute, China).

### 2.8 Yeast two-hybrid assays

The full-length *FlbZIP12* coding sequence was cloned into pGADT7. The full-length coding sequences of *FlSnRK2.2*, *FlSnRK2.3*, and *FlSnRK2.6* were individually cloned into the pGBKT7 vector. All primer sequences are listed in **Table S1**. Protein interaction assays were performed by co-transforming the bait and each prey plasmid into yeast (*Saccharomyces cerevisiae*) strain AH109 according to a previously described protocol (Zhou et al., 2017). Selected positive closes were transferred to selective medium lacking Adenine, Histidine, Leucine, and Tryptophan (Ade, His, Leu, and Trp). Then, yeast cells were incubated at 30°C for 4 days. The empty vectors were co-transformed as negative controls, and the interaction of AtCARK3 and AtRCAR12 was used as a positive control (Wang et al., 2020a).

### 2.9 Bimolecular fluorescence complementation assays

For the BiFC assay, the full-length coding sequences of *FlSnRK2.2*, *FlSnRK2.6*, and *FlbZIP12* without the stop codon were individually cloned into the pSPYCE and pSPYNE vectors, respectively. These vectors were introduced into Agrobacterium strain GV3101. Positive Agrobacterium colonies were incubated overnight in LB medium with the appropriate antibiotics and resuspended in infiltration buffer (0.1 mM acetosyringone, 2 mM Na_3_PO_4_, and 10 mM MES-KOH pH 5.6) to identical concentrations (OD_600 =_ 0.8). The Agrobacterium cells were infiltrated into *N. benthamiana* leaves using a needleless syringe. After 2 days, cells with YFP fluorescence were observed and imaged with a confocal laser-scanning microscope.

## 3 Results

### 3.1 Distribution and morphology of *F. leptopodum*

**Figure 1:**
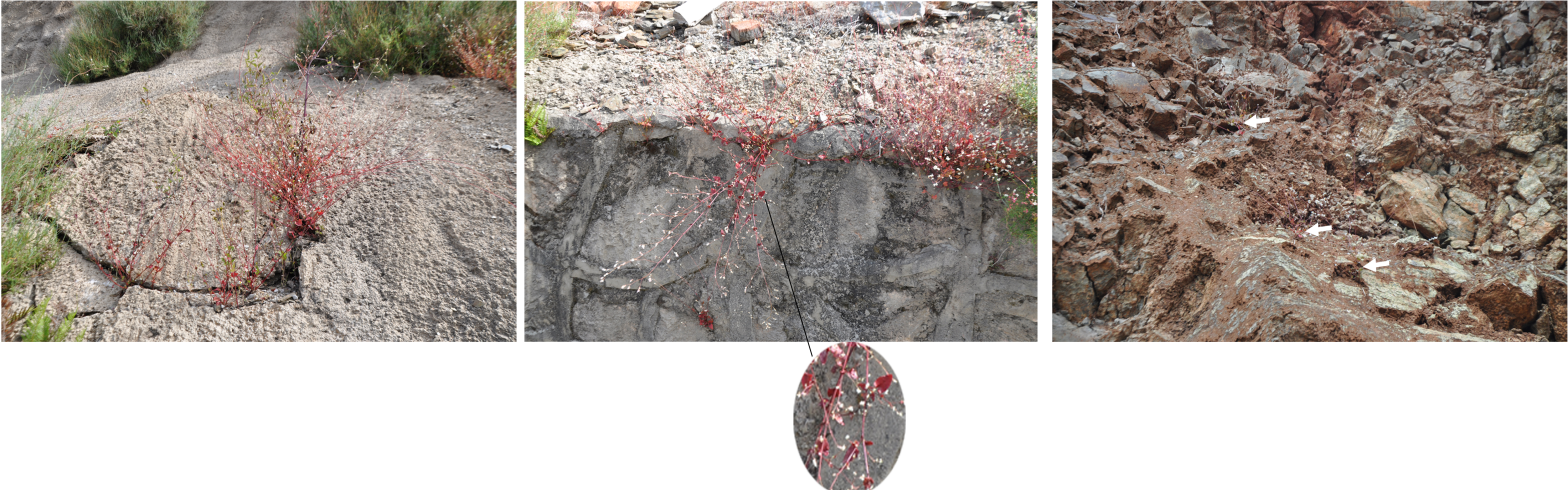
Habitat of *F. leptopodum* in southwestern China (Muli, Sichuan). The white arrows indicate *F. leptopodum*.

**Figure 2:**
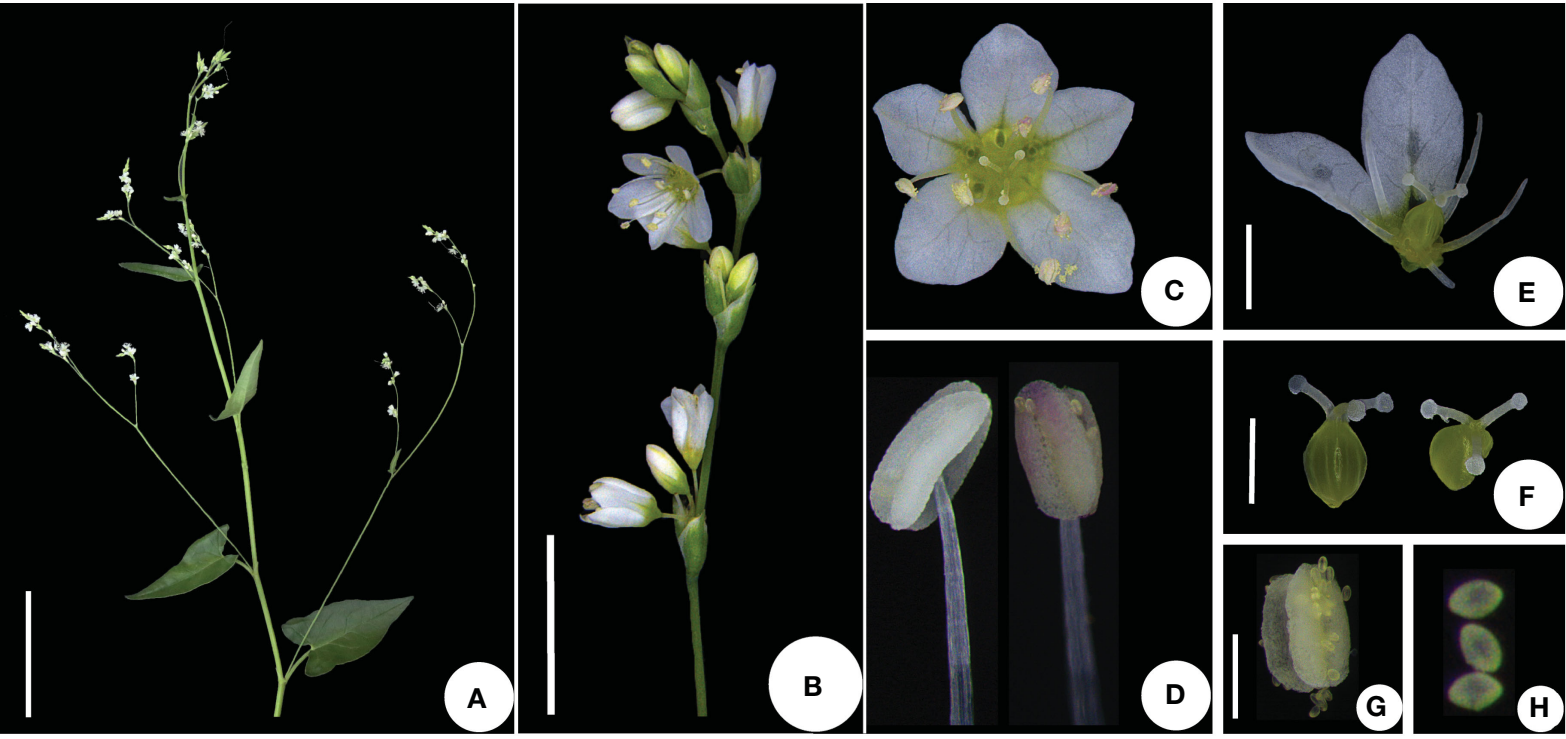
Flower structure of *F leptopodum*. **(A)** Compound raceme; **(B)** raceme; **(C)** mature flower; **(D)** stamen; **(E)** partial structure of a flower (showing a superior ovary); **(F)** pistil (showing the stigma morphology); **(G)** anthers; **(H)** pollens. Scale bars: A, 5 cm; B, 5 mm; D, 2 mm; E, 400 μm; F, 1 mm; G, 400 μm; H, 200 μm; I, 50 μm.

We investigated the distribution of *F. leptopodum* in southwest China, where we discovered only five populations (**Table 1**). The distribution of the species ranges from 888 to 2503 m in altitude, East longitude from 100^°^56′0239″ to 103^°^35′2255″, North longitude from 26^°^16′5844″ to 31^°^30′2127″ (**Table 1**). We noticed morphological differences between plants grown in different habitats (**Figure 1**). For example, we observed deep red leaves for all plants grown in the wild, whereas they developed green leaves in a growth chamber, which is consistent with a previous survey (Fan et al., 2021). According to herbarium records, *F. leptopodum* was previously collected from three sites, where we found no *F. leptopodum* specimens, indicating habitat loss. We did however collect one sample from the buckwheat variety *F. leptopodum* var. *grossii*, exhibiting sparse flowering on racemes, which is consistent with a previous survey (Hu et al., 2016). Similar to the different appearance of leaves, we observed pink, white, or pale-pink flowers on *F. leptopodum* in the field, but these plants produced white flowers when grown in the growth chamber (**Figure 2A**).

We analyzed the inflorescence and flowers of *F. leptopodum* plants germinated from seeds collected at different sites and cultivated on nutrient-rich soil (vermiculite:soil = 3:1). These plants produced dense or sparse panicles (**Figure 2B**). The pistils and stamens were unequal in length in all flowers, indicating that *F. leptopodum* is a cross-pollinating species (**Figure 2C**). Flowers radiated symmetrically, with a calyx composed of five sepals and a simple, five-part perianth (**Figure 2D**). Each flower contained eight stamens, arranged in two whorls, with five stamens in the outer whorl and three stamens in the inner whorl (**Figure 2E**). Each flower also consisted of three styles terminating with a capitate stigma, and mature disc flowers were glandular (**Figure 2F**). The ovary was superior and consisted of three carpels, which were combined into a single-loculed ovary (**Figures 2G, H**).

**Figure 3:**
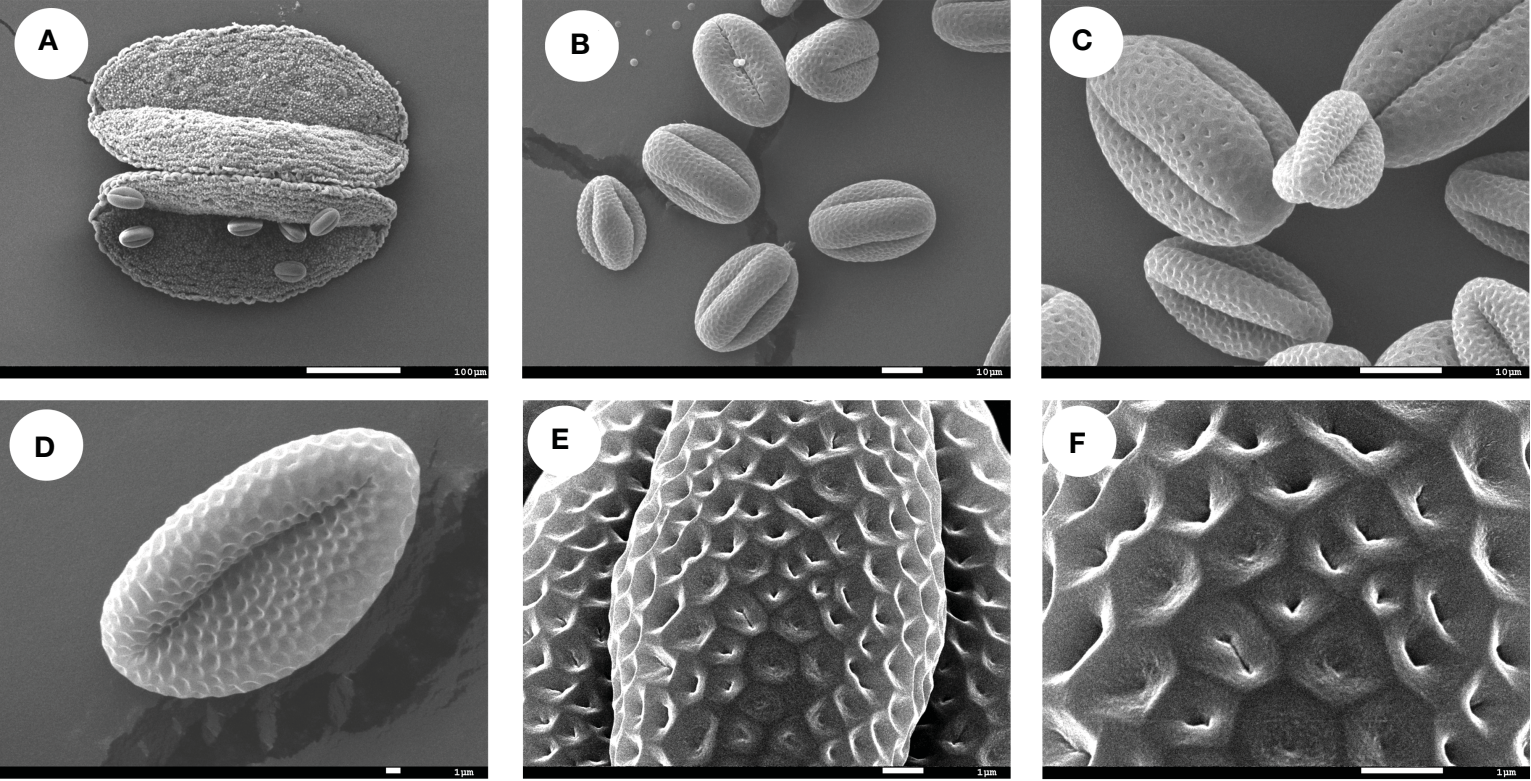
Pollen morphology of *F. leptopodum*. Scale bars: **(A)**, 100 μm; **(B, C)**, 10 μm; **(D–F)**, 1 μm.

We also examined pollen grains by scanning electron microscopy. The pollen grains were spherical or elongated, with three well-defined grooves, but lacking a sulcus (**Figures 3A-F**). The outer wall decoration mesh exhibited edges and corners, some mesh being elongated, and the mesh ridge showed clear peaks (**Figure 3F**).

### 3.2 Overexpressing *FlbZIP12* enhances drought tolerance

**Figure 4:**
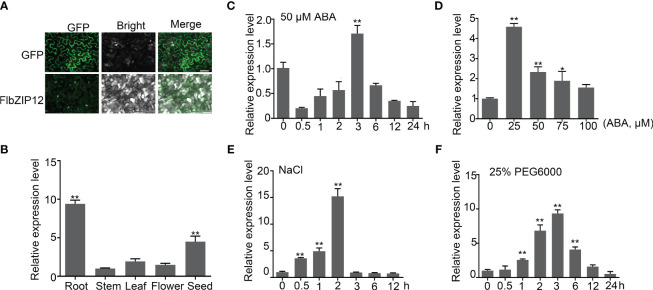
*FlbZIP12* expression is induced by abiotic stresses. **(A)** Localization of FlbZIP12 in *N. benthamiana* epidermal cells. pCAMBIA2300-FlbZIP12-GFP or the empty vector was transiently expressed in *N. benthamiana* cells, and then GFP signals were observed using a Leica fluorescence microscope. At least three leaves were observed. Scale bars, 50 μm. **(B)** Relative *FlbZIP12* expression among different tissues in *F leptopodum* as determined by RT-qPCR. The expression in stems was set to 1. **(C)** Relative *FlbZIP12* expression in 10-d-old seedlings after treatment with 50 μM ABA at the indicated time. **(D-F)** Relative *FlbZIP12* expression induced by various concentrations of ABA for 3 h **(D)**; 200 mM NaCl **(E)**; and 25% PEG6000 **(F)**. *FlH3* was used as an internal control. Values are means ± SD (*n* = 3). Significant differences are indicated (*P < 0.05; **P < 0.01; one-way ANOVA with Tukey’s post-test).

Based on the habitat of *F. leptopodum*, we speculated that it would display strong drought tolerance compared to common buckwheat or Tartary buckwheat. Thus, exploring drought tolerance genes in this species is crucial for improving drought responses in buckwheat. When plants suffer from water scarcity, ABA accumulates to promote multiple physiological responses through the ABA signaling pathway (Leung and Giraudat, 1998; Cutler et al., 2010). ABA-related transcription factors have important roles in ABA signaling transduction (Raghavendra et al., 2010; Zhao et al., 2020). Here, we cloned a gene homologous to ABSCISIC ACID RESPONSIVE ELEMENT-BINDING FACTORs (ABFs) from Arabidopsis. We named FlbZIP12, which displayed high similarity with FtbZIP12 (**Figure S1A, B**). We cloned the *FlbZIP12* coding sequence in-frame and upstream of the green fluorescent protein (*GFP*) sequence and transiently expressed the resulting construct in *N. benthamiana* leaves. The FlbZIP12-GFP fusion protein mainly localized in the nucleus (**Figure 4A**).

We analyzed the *FlbZIP12* expression profile by reverse-transcription quantitative PCR (RT-qPCR): *FlbZIP12* was highly expressed in roots, at lower levels in seeds, and at very low levels in stems (**Figure 4B**). Further, *FlbZIP12* expression was induced in seedlings treated with 20 μM ABA for 3 h, and was upregulated about 2-fold (**Figure 4C, D**). In the presence of 25% polyethylene glycol 6000 (PEG6000) to simulate osmotic stress, *FlbZIP12* expression significantly increased at 1 h into the treatment relative to untreated control seedlings (**Figure 4E**). Seedlings treated with 200 mM NaCl showed a 15-fold increase in *FlbZIP12* expression at 2 h into treatment, compared to untreated control seedlings (**Figure 4F**). These results indicate that FlbZIP12 is involved in abiotic stress.

**Figure 5:**
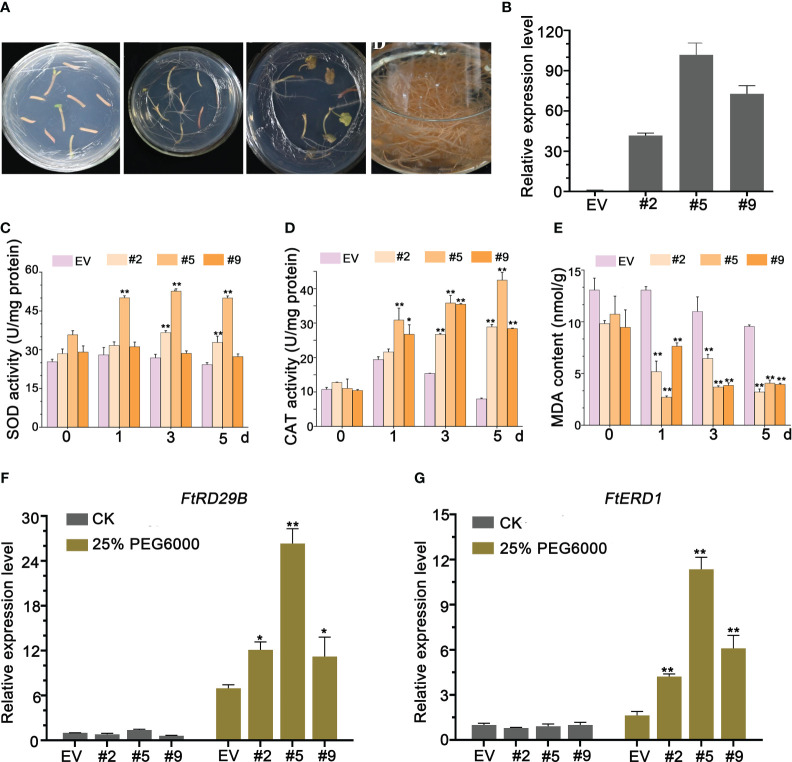
*FlbZIP12* overexpression enhances drought tolerance. **(A)** Transformation of Tartary buckwheat hairy roots. **(B)** Relative *FlbZIP12* expression in 28-d-old hairy root lines (#2, #5, and #9) and control plants (transformed with the empty vector). **(C-E)** Oxidative response after 25% PEG6000 treatment. **(C)** SOD activity; **(D)** CAT activity; **(E)** MDA content. **(F, G)** Relative expression levels of stress-related genes in hairy root lines treated with 25% PEG6000 for 5 d The expression level of *FlbZIP12* in the empty vector control was set to 1 **(B, F, G)**. *FlH3* was used as an internal control **(B, F, G)**. Values are means ± SD (*n* = 3, **B**–**G**). Significant differences between the empty vector (EV) control and *FlbZIP12*-overexpressing hairy root lines are shown (*P < 0.05; **P < 0.01; one-way ANOVA with Tukey’s post-test).

To explore the functions of FlbZIP12 *in planta*, we overexpressed *FlbZIP12-GFP* in Tartary buckwheat hairy roots, yielding three independent lines (#2, #5, and #9) with higher *FlbZIP12* expression levels than the empty vector negative control (pCAMBIA2300-GFP) (**Figure 5A, B**). As these materials are not well suited to perform drought tolerance assays, we tested the effects of simulated drought by treatment with 25% PEG6000.

Reactive oxygen species (ROS) are volatile and short-lived molecules playing important roles in several physiological functions, including acclimation to drought, salinity, or osmotic stresses (Jin et al., 2022). ROS scavenging in plants is mediated by an array of enzymes including superoxide dismutase (SOD) and catalase (CAT) (Gill and Tuteja, 2010; Sewelam et al., 2016). Thus, we measured SOD and CAT activity in transgenic lines treated with PEG6000 for 1, 3, or 5 h, using untreated samples as 0-h controls. *FlbZIP12* overexpression resulted in a significant increase of SOD and CAT activities compared to the control (**Figure 5C, D**). Furthermore, the SOD and CAT activities were strongest in OE#5 plants, which also had the highest *FlbZIP12* expression level among the three lines (**Figure 5C, D**). We also measured the levels of malondialdehyde (MDA), the product of lipid oxidation and a marker of oxidative stress, in these hairy roots. *FlbZIP12* overexpression was associated with lower oxidative damage in response to PEG6000 treatment relative to the control (**Figure 5E**). These data indicate that FlbZIP12 decreases ROS levels, leading to decreased MDA content in response to unsuitable environments in *F. leptopodum*. Similarly, oxidative stress was previously observed in response to water stress in buckwheat species (Aubert et al., 2020).

In addition, FlbZIP12, as a transcription factor, directly or indirectly activates the transcription of stress-responsive genes, including *RESPONSIVE TO DESICCATION29B* (*RD29B*) and *EARLY RESPONSIVE TO DEHYDRATION STRESS1* (*ERD1*). Thus, we performed an RT-qPCR analysis to test mRNA levels for these two genes in our hairy root transgenic lines. After PEG6000 treatment, the *RD29B* and *ERD1* transcript levels were upregulated in the *FlbZIP12* overexpressing lines compared to the negative control line (**Figure 5F, G**). However, there was no significant difference among hairy roots without treatment. There results suggest that FlbZIP12 regulates the expression of stress-related genes in response to drought or osmotic stress.

### 3.3 FlbZIP12 interacts with the kinases FlSnRK2.2 and FlSnRK2.6

**Figure 6:**
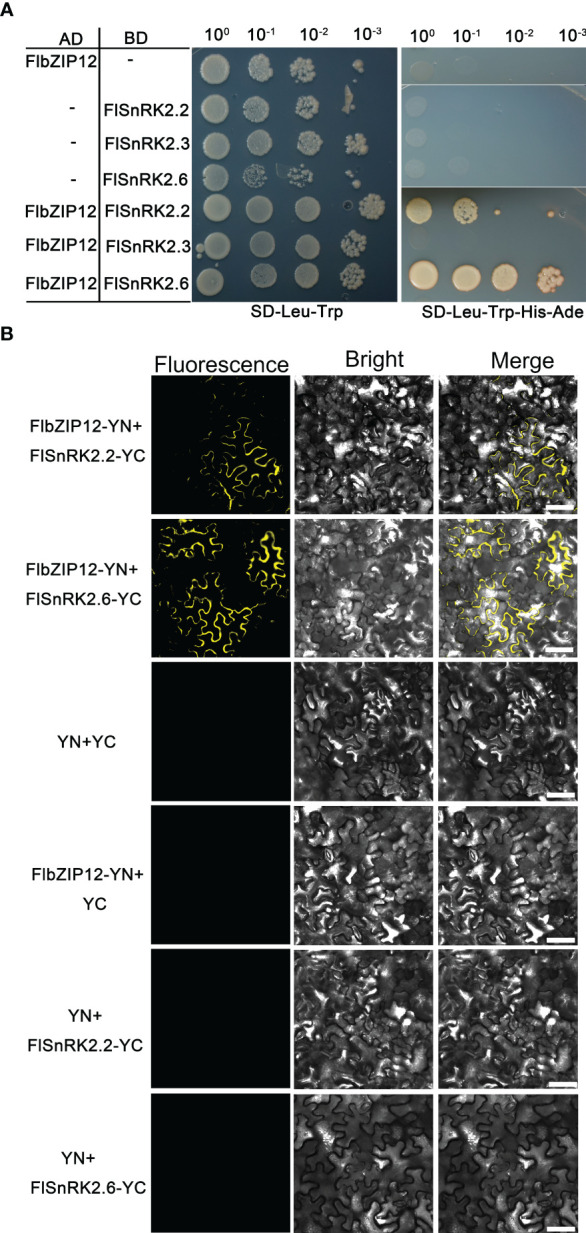
FlbZIP12 interacts with FlSnRK2s. **(A)** FlbZIP12 interacts with FlSnRK2.2 and FlSnRK2.6 in yeast. Yeast cells co-expressing a construct encoding FlbZIP12 fused to the GAL4 AD and a construct encoding FlSnRK2.2, FlSnRK2.3, or FlSnRK2.6 fused to the GAL4 BD were spotted onto synthetic defined (SD) medium lacking Leu and Trp (–Leu–Trp) to select positive transformants and on SD medium –Leu–Trp–His–Ade to test for protein–protein interaction. Growth was monitored after 4 d **(B)** Bimolecular fluorescence complementation assays showing the interactions of FlbZIP12 and SnRK2s in *N. benthamiana* leaves. N‐terminal YFP and C‐terminal YFP were fused to FlbZIP12 or FlSnRK2s, respectively, and the fusion constructs were co‐expressed in *N. benthamiana* leaves followed by observation on a confocal microscope. Scale bar, 30 μm. At least three leaves were observed.

FlbZIP12 shares high sequence similarity to the Tartary buckwheat transcription factor FtbZIP5 (**Figure S1**). In a previous study, FtbZIP5 was shown to interact with FtSnRK2.6 in yeast (*S. cerevisiae*) (Li et al., 2020). Thus, we hypothesized that FlbZIP12 might interact with FlSnRK2s, including FlSnRK2.2, FlSnRK2.3, and FlSnRK2.6. To test this notion, we cloned the coding sequences of several *FlSnRK2*s. We then performed yeast two-hybrid assays using FlbZIP12 as bait: FlbZIP12 interacted with FlSnRK2.2 and FlSnRK2.6, but not with FlSnRK2.3 (**Figure 6A**).

To validate the interaction of FlbZIP12 with FlSnRK2.2 and FlSnRK2.6 *in planta*, we conducted bimolecular fluorescence complementation assays. To this end, we cloned the *FlbZIP12* coding sequence in-frame and upstream of the sequence encoding the N‐terminal half of yellow fluorescent protein (YFP), yielding *FlbZIP12-NYFP*; similarly, we cloned the *FlSnRK2.2* and *FlSnRK2.6* coding sequences in-frame and upstream of the sequence encoding the C‐terminal half of YFP, resulting in *FlSnRK2.2-CYFP* and *FlSnRK2.6-CYFP*. We then transiently expressed the corresponding constructs in *N. benthamiana* leaf cells. We detected fluorescence signals in the cytosol, indicating that FlbZIP12 interacts with FlSnRK2.2 and FlSnRK2.6; by contrast, we detected no fluorescence for the negative control co-expressing the empty vectors *NYFP* and *CYFP* (**Figure 6B**). These finding indicate that the interaction of bZIP-type transcription factors with SnRK2s is conserved in *F. leptopodum*.

### 3.4 FlbZIP12 regulates the flavonoid biosynthesis pathway

**Figure 7:**
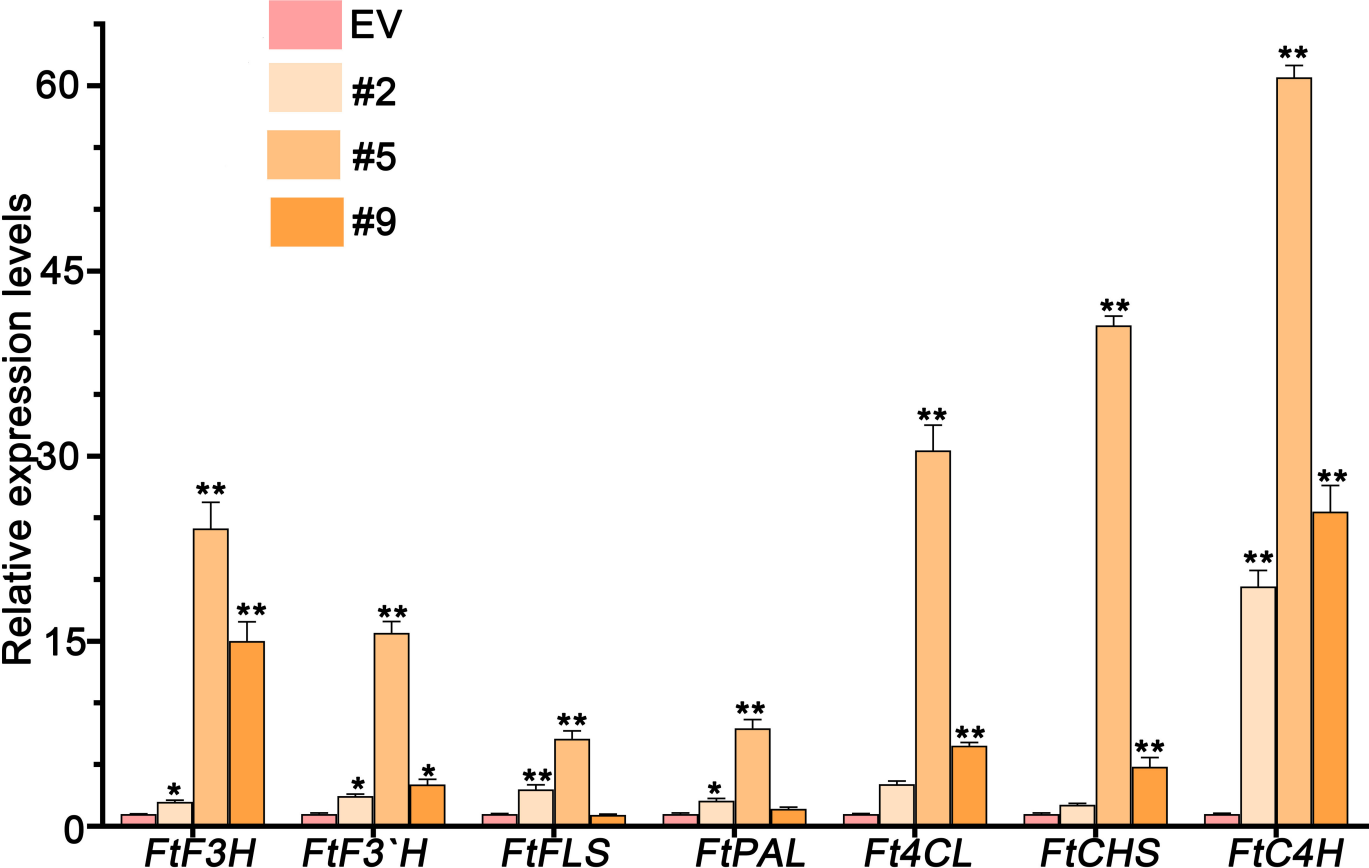
Relative expression levels of flavonoid biosynthesis genes (*FtF3H*, *FtF3′H*, *FtFLS*, *FtPAL*, *Ft4CL*, *FtCHS*, and *FtC4H*) in transgenic hairy root lines and empty vector control (EV) hairy roots. Gene expression levels in the EV roots were set to 1. *FlH3* was used as an internal control. Values are means ± SD of three replicates. Significant differences between the EV control and *FlbZIP12*-overexpressing hairy root lines are shown (*P < 0.05; **P < 0.01; one-way ANOVA with Tukey’s post-test).

We have observed high levels of flavones in plants grown in the wild. Thus, we tested whether FlbZIP12 activates the expression of flavone biosynthesis genes in hairy roots overexpressing *FlbZIP12*. The biosynthesis pathway consists of phenylalanine ammonia-lyase (PAL), followed by reactions catalyzed by cinnamate 4-hydroxylase (C4H), 4-coumarate coenzyme A ligase (4CL), chalcone synthase (CHS), flavone 3-hydroxylase (F3H), flavonoid 3-hydroxylase (F3′H), and flavonol synthase (FLS). All of these flavone biosynthesis genes were significantly more highly expressed in the overexpression line than in the empty vector control line (**Figure 7**). Indeed, *FtC4H* reached 60-fold higher levels in OE#5 hairy roots compared to the empty vector control line (**Figure 7**). These results suggest that FlbZIP12 triggers flavonoid biosynthesis in *F. leptopodum*.

## 4 Discussion

The wild species, *F. leptopodum*, is mainly distributed in water deficit and soil infertile (**Figure 1**). It is suitable for a pioneer species for Karst land or ecological remediation of mining area. For example, Zabelia tyaihyonii is a familiar shrub, growing in the karst forest (Choi et al., 2023). The mining area is polluted by metals, including arsenic, cadmium, and aluminum. Previous studies have shown that Tartary buckwheat combats Al toxicity by rapidly secreting oxalate from roots, externally chelating Al and internally forming nonphytotoxic Al complexes with citrate or oxalate (Wang et al., 2015; Zhu et al., 2015; Zhang et al., 2017). RNA-Seq data indicate that membrane transporters and transcription factors cooperatively response to Al stress (Zhang et al., 2017).

ABA signaling Here, we found that FlbZIP12 interacted with FlSnRK2s (**Figure 6**). SnRK2.2 and SnRK2.3 have been proposed to have a role in the long-term stomatal response to drought conditions (Virlouvet and Fromm, 2015), but are also rapidly activated by ABA and contribute to rapid ABA responses in guard cells (Zhang et al., 2020). The bZIP transcription factors can regulate the transcriptional expressions of stress-related genes by binding specifically to cis-regulatory elements in the promoters, thereby modulating plant stress resistance (Baoxiang et al., 2022). We thus propose that SnRK2.2 and SnRK2.6 activate the bZIP transcription factors, AREB (ABFs), and ABI5 to promote the expression of stress-related genes (Wang et al., 2020b). A previous report showed that FtbZIP83 in Tartary buckwheat also interacts with SnRK2.3 and SnRK2.6 in yeast cells, and overexpressing *FtbZIP85* in Arabidopsis enhanced drought and salt tolerance via the ABA-dependent signaling pathway (Li et al., 2019). In grapevine (*Vitis vinifera*), the bZIP-type transcription factor ABF2 also mediates ROS scavenging ability and cell membrane integrity in response to osmotic stress (Liu et al., 2019). Therefore, FlbZIP12, as a positive regulator, would have the similar function of ABF2 in the ABA signaling pathway in plants.

MYB-bHLH-WD40 (MBW) have been known as the important regulator in flavonoid metabolisms (Hichri et al., 2011). Apart from this, bZIP transcription factors are also involved in flavonoid biosynthesis. In *Ginkgo biloba*, overexpressing *GbbZIP08* in *Nicotiana tabacum* promotes flavonoid accumulation via up-regulating the transcription levels of flavonoid-synthesis-related genes (Han et al., 2023). Here, we found that overexpressing *FlbZIP12* in hairy roots also could upregulate expressions of flavonoid-synthesis-related genes.

Plants respond to drought stress by regulating ABA signal transduction and flavonoid biosynthesis (Gao et al., 2023; Zhang et al., 2023). We showed here that FlbZIP12 may interact with the kinases SnRK2.2 and SnRK2.6 to upregulate *RD29B* and *ERD1* expression to enhance *F. leptopodum* drought tolerance. Moreover, FlbZIP12 may increase flavonoid contents by inducing the expression of flavone biosynthesis genes for acclimation to unsuitable environments. However, more evidence is required on the mechanisms associated with the ABA-dependent signaling pathway in response to drought stress, as well as an understanding of the influence of anthocyanin or rutin biosynthesis.

Phytohormones such as ABA and jasmonic acid play the key roles in inducing cellular signaling pathways related to stress response of plants (Kuromori et al., 2018; Heidari et al., 2020). Many signaling pathways and ion transporters are directly or indirectly induced by ABA in response to abiotic stresses (Faraji et al., 2021; Hashemipetroudi et al., 2023). Besides, bZIP gene family is among the important transcription factors that are induced by upstream factors such as ABA and will control the expression patterns of a large number of target genes.

## Data availability statement

Primers used in this study can be found in the **Supplementary Materials**.

## Author contributions

AW: Investigation, Project administration, Resources, Writing – review & editing. YL: Investigation, Writing – review & editing, Formal Analysis. QL: Investigation, Writing – review & editing. XL: Writing – original draft. XZ: Writing – review & editing. JK: Writing – review & editing. ZL: Data curation, Writing – review & editing. YY: Data curation, Writing – review & editing. JW: Funding acquisition, Project administration, Supervision, Writing – review & editing.
