# Supplementary material for: FlbZIP12 gene enhances drought tolerance via modulating flavonoid biosynthesis in Fagopyrum leptopodum
Source: Front Plant Sci. 2023 Oct 11;14:1279468. doi: 10.3389/fpls.2023.1279468 (PMC10598875; doi:10.3389/fpls.2023.1279468)
Supplement: Supplementary file 1 [file Presentation_1.pptx]

## Slide 1
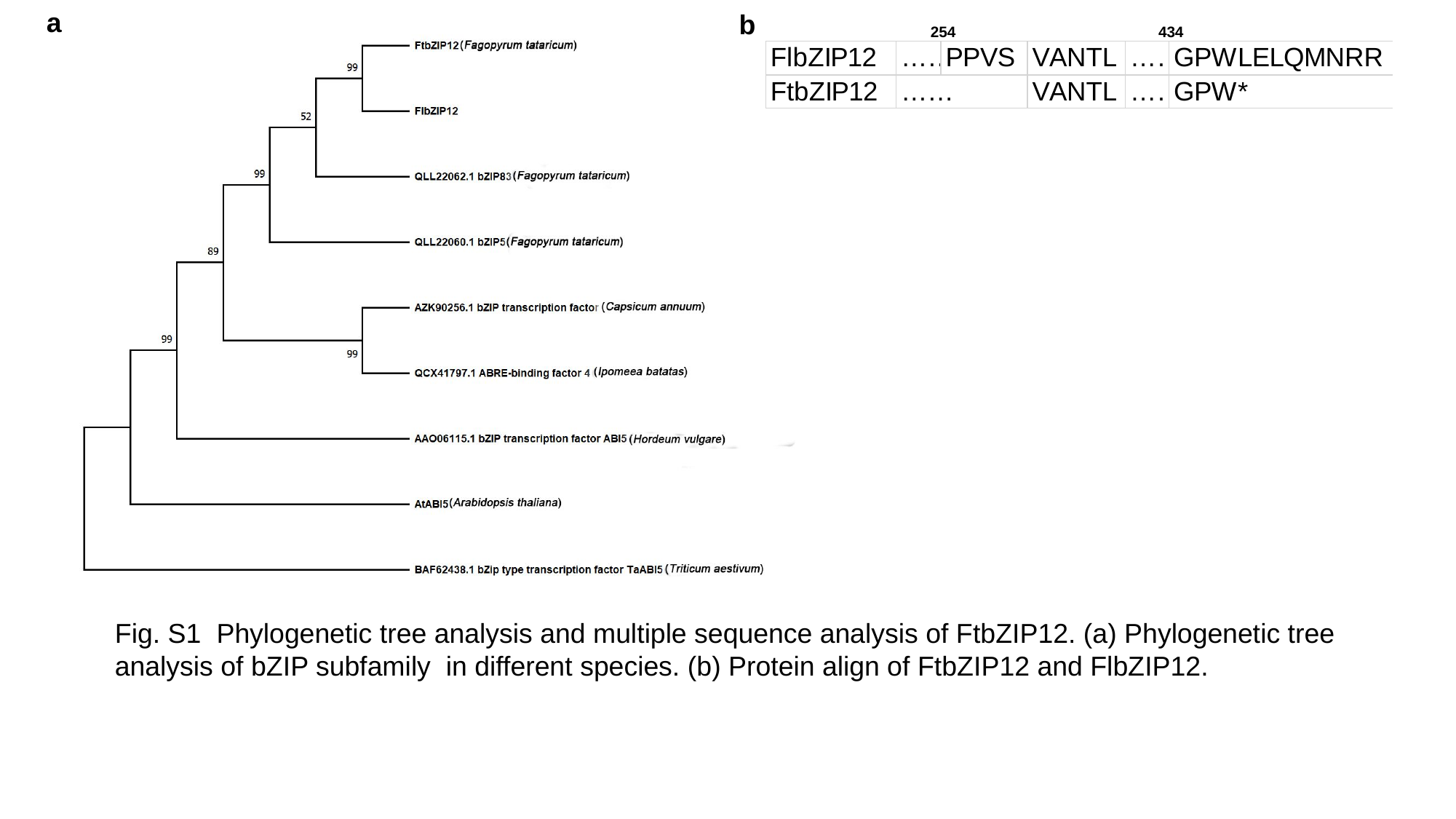

a
b
254
434
Fig. S1 Phylogenetic tree analysis and multiple sequence analysis of FtbZIP12. (a) Phylogenetic tree analysis of bZIP subfamily in different species. (b) Protein align of FtbZIP12 and FlbZIP12.
